# Hospitals for the Psychopathic

**Published:** 1902-11

**Authors:** 


### Hospitals For The Psychopathic.

DURING the last few years the medical profession has been more and more impressed with the fact, that there is a large class of patients for whom there is no proper asylum or abiding place. These patients have physical ailments of such a character that the general hospitals are not necessary to them, and their mental condition is such that the hospitals for the insane, with their legal restrictions, are for many reasons unfit for their care and cure.

This fact has been appreciated and acted upon by Dr. Frederick Peterson, president of the New York Lunacy Commission. On more than one occasion he has advocated the establishment of institutions for the care of neuropathic persons, institutions where the hysterical, the habit cases, the silent cases of epilepsy and other neuropathics may be sent, and where they may be cared for and studied, without legal commitments.

Being without a full knowledge of his views, we recently requested him to write us for publication the needs of such a hospital. In this way only can the subject be brought appropriately and understandingly before the profession.

This is a new step in advance. Shall Buffalo adopt the suggestions of the Commissioner in Lunacy or shall we go on in the same old way sending cases to the hospital for the insane because there is really no other place for them?

We ask for Dr. Peterson's letter thoughtful attention to the end that that subject may be discussed with intelligence at future meetings of our medical societies. It reads as follows:

Office of the State Commission in Lunacy,
Albany, October 15, 1902.

*Editor Buffalo Medical Journal:*

Sir.—I have your letter of October 11 requesting my views as to the need of psychopathic hospitals in the larger cities, and especially as to the expediency of establishing one in Buffalo.

In the first place I assume that all would agree that the sooner a case of insanity, especially an acute one, is put under the best treatment and safest supervision the better. With this idea in view, a sort of emergency hospital, known as the psychopathic hospital has been constructed in a great number of the chief towns in Germany. In many cities, both in this country and Europe, provision has often been made for these emergency cases in connection with general hospitals. Such has been the case for instance in Berlin, Vienna, in Paris, at the Bellevue Hospital in New York and in Philadelphia.

When I was resident physician of the Buffalo General Hospital there was then no asylum in your city and we received emergency cases of insanity at the general hospital, although we had no special provision for them. The methods of commitment are rather slow in this state, and hence many cases of insanity in which treatment should be begun at once have, pending their examination and commitment by legal process, been incarcerated in jails, station houses, or almshouses for some days without treatment. Where the State Asylum is located in the city limits as at Buffalo and Rochester, the need for a psychopathic hospital is not so apparent as in places like Syracuse and Albany, which are remote from any state hospital. At the same time I have been informed that even in Buffalo, patients are not infrequently kept in station-houses awaiting the necessary legal examination and commitment. It is a fact that patients are sometimes sent to hospitals for the insane who are not really insane, but suffering from various forms of delirium due to typhoid fever, pneumonia and other diseases.

I would say that Albany has already established a reception pavilion in connection with the Albany General Hospital, where all these emergency cases of insanity and cases of apparent insanity are now received. This has proved very successful. If there were such a pavilion in connection with some general hospital in Buffalo, these cases could be received and treated immediately, pending their examination and commitment, and in cases which are doubtful their insanity verified before resorting to the legal process.

Let me call your attention to the statistics of the pavilion for the insane at Bellevue Hospital for the five months ending June 30, 1902. There were admitted 986 patients. Of these 652 were sent to the Manhattan State Hospital; 21 to private asylums for the insane; 33 to other institutions; 79 were sent into Bellevue Hospital as not insane, but suffering from other ailments; 182 were sent back to their friends without commitment; 27 died; 24 remained in the pavilion. What I would especially call your attention to here is the remarkable fact that out of this total number of 986 admitted, 79 were sent into the Bellevue Hospital as not insane, and 182 were sent back to their friends; 27 were so ill that they died in the reception hospital. In their classification of diseases received at the pavilion for the insane for three months ending June 30, 1902, there were 584 cases. Among these were 118 cases of epilepsy, hysteria, chorea, alcoholism, morphinism, and the like, and 28 cases marked as not insane. These satistics afford something of a commentary upon the usefulness of such a reception hospital.

When in Buffalo not long ago, I noticed that the newspapers were agitated as to the proper disposition of alcoholic patients who seem by the accounts given to be delirious but not insane and are sent to the penitentiary for treatment. While I admit that the need for a psychopathic hospital in Buffalo, owing to the presence of a large state hospital in your city limits is not so pressing as in other cities, still I believe that there is a place for a reception pavilion of the kind indicated in connection with some one of your hospitals in the central part of the city. FREDERICK PETERSON.

We have called up this important question in this manner, because it deserves patient consideration in the best light that can be obtained, and no one can speak more intelligently in regard to it than the distinguished president of the lunacy commission. It would be well if physicians in Buffalo, and especially our neurologists, would give this matter early and thoughtful study. We should be glad to have it discussed in the columns of the JOURNAL.

